# STAT3 in Skeletal Muscle Function and Disorders

**DOI:** 10.3390/ijms19082265

**Published:** 2018-08-02

**Authors:** Eleonora Guadagnin, Davi Mázala, Yi-Wen Chen

**Affiliations:** 1 Department of Orthopeadic Surgery, Brigham and Women’s Hospital, Harvard Medical School, Boston, MA 02115, USA; eguadagnin@bwh.harvard.edu; 2 Center for Genetic Medicine Research, Children’s National Health System, Washington, DC 20010, USA; dmazala@childrensnational.org; 3 Department Genomics and Precision Medicine, George Washington University, Washington, DC 20052, USA

**Keywords:** STAT3, hypertrophy, atrophy, IL6, TGF, muscle, satellite cells

## Abstract

Signal transducer and activator of transcription 3 (STAT3) signaling plays critical roles in regulating skeletal muscle mass, repair, and diseases. In this review, we discuss the upstream activators of STAT3 in skeletal muscles, with a focus on interleukin 6 (IL6) and transforming growth factor beta 1 (TGF-β1). We will also discuss the double-edged effect of STAT3 activation in the muscles, including the role of STAT3 signaling in muscle hypertrophy induced by exercise training or muscle wasting in cachectic diseases and muscular dystrophies. STAT3 is a critical regulator of satellite cell self-renewal after muscle injury. STAT3 knock out affects satellite cell myogenic progression by impairing proliferation and inducing premature differentiation. Recent studies in STAT3 signaling demonstrated its direct role in controlling myogenic capacity of myoblasts and satellite cells, as well as the potential benefit in using STAT3 inhibitors to treat muscle diseases. However, prolonged STAT3 activation in muscles has been shown to be responsible for muscle wasting by activating protein degradation pathways. It is important to balance the extent of STAT3 activation and the duration and location (cell types) of the STAT3 signaling when developing therapeutic interventions. STAT3 signaling in other tissues and organs that can directly or indirectly affects skeletal muscle health are also discussed.

## 1. Introduction

Skeletal muscle is a highly plastic tissue that can change mass, function, and metabolism in response to both endogenous and/or exogenous stimuli, which is important in maintaining muscle homeostasis and overall good health. Skeletal muscle homeostasis is achieved by finely balancing catabolic and anabolic processes. Anabolic processes can be modulated by growth factors, nutrient availability, and physical activities, which culminate in the activation of pathways responsible for protein synthesis, including the Insulin Growth Factor 1 (IGF1)/Phosohoinositide 3-kinase (PI3K)/protein kinase B (AKT)/mechanistic target of rapamycin (mTOR) signaling pathway [1,2]. However, disuse and pathological conditions, such as best rest, cast immobilization, muscular dystrophies, diabetes, cancer, kidney and heart diseases can compromise the fine homeostatic balance. The balance shifts towards catabolic processes that lead to a loss of skeletal muscle mass by increasing protein degradation via ubiquitin-proteasome and the autophagy-lysosome systems, which compromises the overall quality of life of affected individuals and leads to increased hospitalization, morbidity, and mortality.

**Figure 1:**
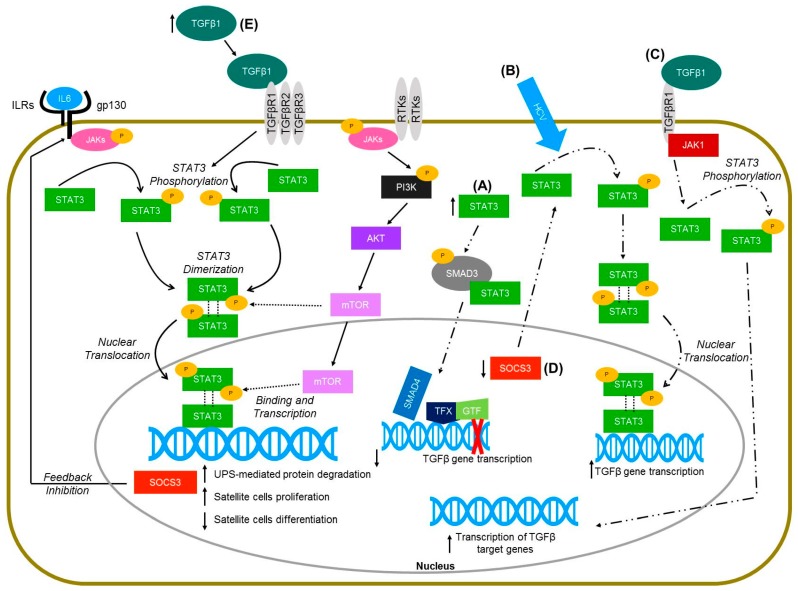
Molecular pathways of STAT3 signaling in skeletal muscle (solid lines) and non-muscle cells (hepatocytes and keratinocytes—dashed lines). (**A**) Increased levels of STAT3 in HaCaT cells lead to the physical interaction of STAT3 with SMAD3, thus inhibiting TGFbeta gene transcription (Wang et al., 2016 [115]). (**B**) Hepatitis C virus infected hetapoma cells have greater activation of STAT3 and subsequent increased TGFbeta gene expression (Presser et al., 2013 [117]). (**C**) TGFbeta activation in hepatic stellate cells leads to janus kinase 1 (JAK1) binding to TGFbeta receptor type 1 (TGFβR1), which leads to the phosphorylation of STAT3 and ultimately the transcription of TGFbeta target genes (Tang et al., 2017 [118]). (**D**) Deletion of the suppressor of cytokine signaling-3 (SOCS3) gene in mice injected with dimethylnitrosamine resulted in increased activation of STAT3 in liver cells, and further increased the expression of TGFbeta 1 (Ogata et al., 2006 [112]). (**E**) TGF-β1 overexpressing mice have increased levels of pSTAT3 (Guadagnin et al., 2015 [5]).

There are a large number of molecular pathways that contribute to muscle growth and wasting processes. Signal transducer and activator of transcription 3 (STAT3) signaling has been known to play a critical role in muscle wasting induced by the interleukin 6 (IL6)/Janus kinase (JAK)/STAT3 signaling pathway [3,4]. Recent studies have shown that transforming growth factor β 1 (TGF-β1) plays a significant role in the progression of muscle diseases and that STAT3 activation exacerbates disease phenotypes induced by TGF-β1 [5]. STAT3 was first identified as a transcription activator that interacts with IL6-responsive elements of acute phase response genes in hepatocytes [6]. STAT3 is expressed at a low, basal level in virtually all the cells. A variety of stimuli can activate STAT3 signaling via protein phosphorylation. Once activated, pSTAT3 dimerizes and enters the nucleus to activate transcription of various genes (Figure 1). STAT3 signaling has been shown to be activated in skeletal muscle and promotes skeletal muscle atrophy in muscle diseases, such as Duchenne muscular dystrophy (DMD), and Merosin-deficient congenital muscular dystrophy (MDC1A), as well as cancer and sepsis [7,8,9]. In addition to skeletal muscles, STAT3 signaling has been shown to play critical roles in both vascular and central nervous systems, thus directly and indirectly contributing to muscle health [10,11,12,13].

The aim of this review is to summarize the current knowledge of STAT3 signaling with a special focus on its role in skeletal muscle health and diseases. We will focus on the cellular and molecular mechanisms linking STAT3 activation and skeletal muscle growth and wasting, and the IL6/STAT3-dependent inhibition of the skeletal muscle regenerative processes.

## 2. STAT3 Modulates Satellite Cell Myogenic Capacity and Self-Renewal

Satellite cells were first discovered over 50 years ago as mononucleated cells residing peripherally on skeletal muscle fibers [14]. Subsequent experiments demonstrated that satellite cells lie quiescent on the muscle membrane, but once activated, following muscle injury, have the ability to undergo mitosis [15,16,17]. Activated satellite cells undergo proliferation, differentiation, and eventually fuse with existing myofibers or form newer myofibers. The activation and subsequent progression of satellite cells into the myogenic lineage is governed by several different transcription factors (e.g., Pair box 7 (Pax7), myogenic factor 5 (Myf5), myoblast determination protein (MyoD), and myogenin), and signaling pathways (e.g., Wnt and Notch) [18]. Although multiple studies have shown the effects of STAT3 on the myogenic lineage of myoblasts in culture, only a limited number of studies have evaluated the role of STAT3 in adult muscle satellite cells.

STAT3 has been shown to be activated in Pax7+ satellite cells after acute injury while satellite cells engage in tissue repair [19]. Moreover, phosphorylated STAT3 (pSTAT3) was detected in satellite cells after 4 days in culture, and this correlates with the expression of MyoD, suggesting that STAT3 might play a role on the fate of satellite cells [19]. To demonstrate the role of STAT3 in satellite cell myogenic progression, satellite cells were infected with a lentivirus expressing shRNA against STAT3 and the expression of MyoD and myogenin was evaluated [19]. Knockdown of STAT3 in satellite cells decreased the expression of both MyoD and myogenin, thus demonstrating the pivotal role of STAT3 in satellite cell myogenic progression in vitro [19]. To further evaluate if the in vitro experiments would yield similar results in vivo, STAT3 was conditionally ablated in Pax7-expressing satellite cells in 2-month-old *Pax7-CreER*; Stat3flox/flox mice [19]. In vivo STAT3 deletion increased the number of Pax7+ satellite cells in muscles 30 days after tamoxifen treatment and also led to an increased number of Pax7+ satellite cells after skeletal muscle injury [19]. Nevertheless, muscle regeneration was altered, as shown by smaller myofiber size 25 days post-injury, which suggested an important role of STAT3 for myogenic differentiation in vivo [19]. Lastly, transient delivery of a STAT3 inhibitor in vivo improved muscle regeneration after injury in both young and older mice [19]. Overall, these findings demonstrate that normal STAT3 activity is required for proper muscle maintenance, yet intermittent STAT3 inhibition could have promising implications to augment muscle regeneration in muscle wasting diseases.

Levels of pSTAT3 in satellite cells increases in muscles from younger to older healthy wildtype (WT) mice [20]. More specifically, pSTAT3 protein levels were 1.6- and 2.4-fold higher in satellite cells from young (2–4 months) and older adult (16–20 months) mice compared to satellite cells from adolescent mice (3 weeks), respectively. Satellite cells treated with a siRNA against STAT3 had no change in the number of total dividing cells compared to controls, but demonstrated a 2-fold increase in symmetric cell division [20]. To evaluate the role of STAT3 in satellite cell engraftment, FACS isolated quiescent Pax7-ZsGreen satellite cells were transfected with siSTAT3 and then transplanted into cardiotoxin injured tibialis anterior (TA) muscles from WT mice [20]. Engraftment of Pax7+ and ZsGreen double-positive cells were assessed 12 days post-transplantation, and results demonstrated that knockdown of STAT3 significantly increased the repopulating capacity compared to control siRNA (scramble) injected tibialis anterior (TA) muscles [20]. In vivo STAT3 inhibition also enhanced muscle regeneration. Briefly, STAT3 pharmacological inhibition in cardiotoxin injected TA muscles increased fiber size, decreased the number of developmental myosin heavy chain positive fibers, and decreased macrophage infiltration [20]. While it is not clear whether the in vivo effects were solely due to STAT3 suppression in satellite cells, since myofibers and other cell types may have also been potentially affected by these approaches, the findings demonstrated that STAT3 plays an important role in satellite cell function and muscle regeneration.

The chemical inhibitors and siRNA used in the studies above could have off-target effects, thus limiting the interpretation that their results were only due to the effects of STAT3 in muscle satellite cells [21]. Moreover, since STAT3 is ubiquitously expressed, STAT3 inhibitors could have influenced muscle regeneration due to the effects of STAT3 inhibition in different cell types and not only due to its effect on satellite cells [21]. To better address the role of STAT3 in adult muscle satellite cells, a mouse model with a conditional deletion of STAT3 only in muscle satellite cells was generated. Conditional knockdown of STAT3 in muscle satellite cells did not affect normal muscle development or the number of Pax7+ satellite cells in muscles from STAT3 knockout (KO) mice compared to WT mice [21]. To evaluate the effects of muscle satellite cell STAT3 KO in muscle regeneration, different muscles were injured with cardiotoxin [21]. Cardiotoxin injected muscles from STAT3 KO mice only demonstrated a defect in muscle regeneration 5 days post-injury, as shown by increased degeneration and fewer regenerated fibers at the site of injury [21]. Moreover, cardiotoxin injured muscles from STAT3 KO mice had 50% fewer Pax7+ satellite cells compared to muscles from WT mice, which suggested that STAT3 plays a critical role in satellite cell self-renewal [21]. Muscles were sequentially injected with cardiotoxin, one week apart, to further evaluate the role of STAT3 in muscles undergoing repeated cycles of degeneration. Injected muscles from STAT3 KO mice showed a greater deficit in muscle regeneration after repeated cardiotoxin injections, as shown by a greater area of degeneration without repair and increased embryonic myosin heavy chain in the surrounding areas [21]. Yet, sequentially injured muscles from STAT3 KO fully repaired by 30 days post-injury with only a deficit in the number of Pax7+ satellite cells compared to WT mice [21]. The effects of STAT3 conditional knockdown were also evaluated in vitro. In vitro experiments demonstrated that STAT3 KO satellite cells have defective proliferation and increased susceptibility for premature differentiation [21]. According to the authors, changes in the rate of proliferation and differentiation in STAT3 KO satellite cells could be due to downregulation of STAT3 target genes, such as Pax7, Igf2bp2, and Hmga1 [21]. Collectively, these results suggest that STAT3 is a critical regulator of satellite cell self-renewal after muscle injury, and that STAT3 KO affects satellite cell myogenic progression by impairing proliferation and inducing premature differentiation.

## 3. STAT3 Signaling in Response to Physiological Stimuli

Physical activity and exercise training are important for maintaining muscle mass and function. In addition, healthy skeletal muscles and adequate physical activity levels are essential to general health and maintaining metabolic homeostasis. Previous studies showed that IL6 upregulation and STAT3 activation are essential components for the adaptive responses to resistance training [22,23,24]. The IL6 and STAT3 activation was also shown to be directly associated with increased glucose transport in both in vivo and ex vivo experiments [25]. For instance, vastus lateralis muscle strips from healthy men, incubated with IL6 (120 ng/mL), had greater glucose transport, glucose incorporation into glycogen, and glucose oxidation compared to control samples.

An acute or a single bout of exercise and muscle damage activates STAT3 in satellite cells, which promote satellite cell proliferation and migration. Therefore transient STAT3 activation in muscles is believed to be beneficial to muscle regeneration and hypertrophy. However, a recent study showed that STAT3 activation was not required for exercise induced muscle hypertrophy in human muscle biopsies obtained after resistance exercises and synergist ablation in the STAT3 KO mice [26]. The study showed that pSTAT3 muscle levels from individuals undergoing two types of resistance exercises were not changed pre- vs. post-exercise. In addition, both WT and STAT3 KO mice had similar muscle hypertrophy after synergist ablation. The finding from the study highlights the importance of the type and intensity of stimuli, the timing of the stimuli in the activation of STAT3 signaling, and the cell types where the STAT3 activation occurs [27]. More studies are needed to carefully define these factors. In addition to myofibers and satellite cells, activation of STAT3 in immune cells in the muscles may also play significant roles in regulating muscle responses to exercise training and stimuli that lead to muscle wasting [28]. Extensive studies have been done to investigate the benefit of exercise training to different diseases, including obesity, diabetes, and cancer cachexia [29,30,31,32,33,34]. Several studies showed that STAT3 activation contributes to central leptin signaling induced by exercise, which has metabolic benefit [35,36,37]. Endurance training has been shown to increase leptin sensitivity in skeletal muscles [35,38].

While IL6, induced by high intensity exercises, and STAT3 activation have been shown to promote muscle hypertrophy, chronic IL6 elevation and STAT3 activation have been shown to be involved in muscle wasting [4,5,39,40,41]. TA muscles chronically infused with IL6 for 14 days had increased phosphorylation of STAT3 and activation of its downstream target suppressor of cytokine signaling 3 (SOCS3) [39]. Recent studies showed that STAT3 signaling contributes to muscle wasting via different upstream activators, including IL6, interferon gamma (IFNγ)/tumor necrosis factor alpha (TNFα), and TGF-β1 [4,5,40,42]. The pSTAT3 then directly and indirectly activates downstream genes, including Myod1, which is the master regulator of myogenesis, and SOCS3, which negatively regulate STAT3. Table 1 listed known STAT3 regulated genes in skeletal muscles. STAT3 activation also has been shown to be responsible for the activation of caspase and ubiquitin-proteasome system, therefore, the inhibiting of STAT3 signaling preserves muscles mass [4,41,42,43,44]. In addition to STAT3 activation in muscles, muscle wasting can be caused by denervation, which can be affected by the nerve regeneration capacity. STAT3 has been shown to be activated in both central and peripheral nerve injuries [11,45]. STAT3 is important for motor hippocampal neuron differentiation [29,45]. Axonal STAT3 is activated by the injury and provides a retrograde signaling to the nucleus, which promotes regeneration of both sensory and motor neurons [45,46,47,48,49].

## 4. STAT3 Signaling Contributes to Muscle Wasting in Cancer Cachexia

The IL6/STAT3 signaling pathway has been implicated in cancer cachexia, a multifactorial condition that presents with skeletal muscle wasting, adipose tissue atrophy, and anorexia. Cachexia is defined by the loss of 5–10% or more in body weight associated with cancer or other inflammatory conditions [63,64]. The prevalence of cachexia accounts roughly for 80% of upper gastrointestinal cancer patients and 60% of lung cancer patients at the time of diagnosis. Cachexia correlates with diminished quality of life and a high mortality rate in cancer patients [65].

Physiological changes associated with cancer cachexia are anorexia, inflammation, insulin resistance, and, more importantly, an increase in the breakdown of muscle proteins. The muscle wasting process is triggered by an increase in muscle protein catabolism leading to a net loss of muscle mass. One of the major contributors to the proteolytic process is the proteasome-ubiquitin pathway. However, a further concurring factor is the increase in resting energy expenditure due to altered mitochondria dynamics, which would cause the uncoupling of respiration from adenosine triphosphate (ATP) production, leading to dissipation of energy as heat [66].

The phenotypic characteristics of cancer cachexia are well recapitulated in several animal models, including the colon cancer model Apc^Min/+^, the Kras-induced lung cancer mouse, Lewis lung carcinoma, C26 adenocarcinoma, and B16 melanoma, where the cachexia level is dependent upon the increasing level of circulating IL6 [67,68,69]. In vivo evidence of the specific involvement of IL6/STAT3 in causing cachexia-associated muscle wasting is that Apc^Min/+^ mice lacking IL6 do not develop cachexia [3,42]. Moreover, systemic overexpression of IL6 in cancer-free mice or pre-cachectic Apc^Min/+^ mice accelerates the onset of cachexia [67]. Circulating levels of IL6 in Apc^Min/+^ mice were significantly correlated with Apc^Min/+^ mouse body weight, and with the level of STAT3 phosphorylation in slow oxidative and fast glycolytic skeletal muscles [70]. IL6 plasma levels have also been examined in cancer patients, and they were found to be variably but consistently elevated in cancer patients compared to healthy controls [71,72,73,74]. Moreover, plasma levels of IL6 were significantly correlated to the degree of tumor progression and symptoms of cachexia (loss of lean mass, severe fatigue, and anorexia) in humans. However, the studies limited their analysis to serum IL6 and did not analyze total or activated STAT3 protein, in either skeletal muscle or other tissues.

The IL6/STAT3 pathway is thought to induce muscle wasting in cachexia experimental models via two routes. STAT3 phosphorylation is necessary to activate the acute phase response protein gene expression in the liver, which is a generalized response to insults (i.e., cancer) by the innate immune system [75]. Cancer cachexia has been linked to persistent activation of the acute phase response, and skeletal muscle is currently considered the primary source of protein to produce acute phase response proteins [76,77]. It is estimated that 2.6 g of muscle protein is needed to be mobilized to synthesize 1 g of acute phase response proteins. Skeletal muscles of C26 tumor-bearing mice were used to elucidate the connection between the elevated IL6/STAT3 signaling, cancer cachexia, and muscle wasting [44,78]. The data showed a predominant STAT3 transcriptional signature which correlated with increased expression of skeletal muscle ubiquitin E3 ligases Murf-1 and Atrogin-1, likely responsible for skeletal muscle atrophy. In addition, the STAT3 activation was responsible for the upregulation of many of the acute phase response genes. Inhibiting STAT3 using a mutated STAT3 construct to induce dominant negative activity is sufficient to abrogate the skeletal muscle wasting downstream of IL6 partly by inhibiting the activity of the ubiquitin proteasome system, in vitro and in vivo [42]. The activation of the ubiquitin-proteasome system by STAT3 could be direct, via direct binding of STAT3 to ubiquitin proteasome system (UPS)-related gene’s promoters, or indirect, via caspase-3 activation, which can independently drive proteolysis or cleave subunits of the 19 S proteasome and activate the proteolytic activity of the 26 S proteasome. A contributing factor to skeletal muscle wasting is the decrease protein synthesis due to the AMP-activated kinase (AMPK)-dependent repression of mTOR signaling, which is most evident in the later stages of cancer cachexia [78].

Another aspect of skeletal muscle metabolism, regulated by IL6/STAT3 pathway in cancer cachexia, is the process of muscle repair via satellite cells recruitment. It is now known that skeletal muscles of cachectic mice are subjected to damaging stimuli due to the circulating cytokines produced by tumor and host-defense, in particular TNFα, INFγ and IL6. The inflammatory responses promote satellite cell proliferation but not cell differentiation. The proliferating progenitor muscle cells in the cachectic environment were shown consistently expressing Pax7 (the marker for self-renewal satellite cells) but not differentiation factors such as myogenin, which compromised the overall regenerative capacity [79,80]. While its specific role in the pathology of cancer cachexia has not been elucidated yet, it is known that STAT3 promotes expansion of the satellite cells pool in vivo and in vitro, and may play a negative role in promoting differentiation and muscle repair [24,81]. In addition, recent studies have suggested that STAT3 might act by downregulating mTOR and p70S6K activity, two downstream effectors of the AKT signaling pathway involved in muscle differentiation, independently of AKT activation [81].

## 5. STAT3 Signaling in Inflammatory Myopathies

The most well documented function of STAT3 has been linked to its role on the immune system. The purpose of the immune system is to protect the body against pathogens, but disorders in the immune system can lead to conditions such as autoimmune diseases and cancer [82]. STAT3 mutations have been associated with immunodeficiency, autoimmunity, and cancer. Moreover, genomic variations in the STAT3 have been linked to an increased predisposition to diseases such as psoriasis, multiple sclerosis, as well as inflammatory bowel disease [83,84,85,86]. Overall, STAT3 function needs tight control for optimal health since either hyperactivation or inactivation of STAT3 results in human diseases [82].

Inflammatory myopathies are thought to be autoimmune diseases characterized by chronic muscle inflammation and muscle weakness. They include diseases such as polymyositis (PM), dermatomyositis (DM), sporadic inclusion body myositis, myositis associated with cancers, overlap myositis, and immune-mediated necrotizing myopathies [87]. Interleukin 22 (IL22) is a cytokine either playing a protective or a pathological role in different autoimmune diseases [88,89]. Once bound to its receptor, IL22 induces activation of STAT3, thus initiating an inflammatory response [90]. Affected muscles from PM/DM patients have increased activation of the IL22 pathway [88]. Briefly, IL22 protein levels were significantly upregulated in muscles from PM/DM patients and correlated with disease severity [88]. Moreover, levels of IL22 binding protein (IL22BP), a natural inhibitor of IL22, were not altered, which suggested a potential imbalance between IL22 and IL22BP in muscles from PM/DM patients [88]. Although STAT3 levels were not altered in muscles from PM/DM patients, the levels of pSTAT3 were higher in affected muscles from those patients [88]. Lastly, pSTAT3 was mostly found in cells expressing the IL22 receptor 1, which suggested that an autocrine inflammatory loop is present in affected muscles from PM/DM patients [88].

In a different study, a patient with a known STAT3 mutation, high levels of immunoglobulin-E, history of multiple infections, fractures, and many other health problems reported to a doctor’s office complaining of pain on the right lower leg [91]. According to the patient, the leg pain and swelling persisted for 3 days and did not affect his ability to ambulate, but the patient reported having to intake oral narcotics to continue with his work due to the severity of pain [91]. An magnetic resonance imaging (MRI) scanning reveled inflammation of the peroneus brevis and longus muscle, and the patient’s serum creatine kinase (CK) levels were also elevated [91]. After being referred to an infectious disease specialist, the patient was treated with antibiotics (vancomycin and cefepime) for a presumed bacterial myositis [91]. The antimicrobial treatment improved the patient’s symptoms dramatically within 24 h, and the doctors suspected this was caused by small excoriations on the toes on the same side of the pain. Although STAT3 mutations have been associated with skin, lung, and liver infections, isolated infections of muscles are far less common. This case report of an unusual myositis in a patient with a known STAT3 mutation shows that STAT3 immunodeficient patients should be carefully evaluated for proper diagnosis. STAT3 has been shown to play an important part in autoimmune diseases. The studies presented here demonstrate that STAT3 can also play a role in myopathies. While one study demonstrated that pSTAT3 levels correlated with the extent of muscle damage in DM/PM patients, the second study showed an example of a patient with a known STAT3 mutation who developed a bacterial myositis. Further studies will be needed to evaluate if STAT3 also plays a role in different inflammatory myopathies and if other patients with STAT3 mutations develop a muscle phenotype.

## 6. STAT3 Activation in Muscular Dystrophies

Muscular dystrophies (MD) are genetic diseases characterized by progressive a loss of muscle mass and muscle weakness. There are over 30 types of MD caused by mutations in different genes, and while some MD show symptoms in early childhood, other types might not display symptoms until middle age or later in life. Although the genetic cause of most of the MD has been determined, the underlying mechanisms behind disease progression are far more complex and still not completely understood. Thus, studies providing novel disease altering mechanisms that could potentially become therapeutic options are still of relevance. Although STAT3 has been shown to play a role in different muscle wasting conditions (e.g., cancer cachexia and auto-immune disorders), only a limited number of studies investigated its role in MD.

Interleukin 6 (IL6) is a cytokine with pleiotropic functions ranging from the positive regulation of muscle homeostasis, via the regulation of muscle stem cells for muscle hypertrophy, to the promotion of muscle atrophy and wasting in pathological conditions [92]. IL6 has been shown to play a role in Duchenne muscular dystrophy (DMD), one of the most common types of MD. Briefly, steroid-naïve DMD patients had higher serum levels of IL6 compared to glucocorticoid-treated patients [93]. Moreover, immunofluorescence staining demonstrated that IL6 was localized in the interstitial space in infiltrating cells near muscle fibers from steroid-naïve DMD patients [93]. Levels of IL6 were also evaluated in *mdx* mice, the most commonly used mouse model of DMD. IL6 levels were up-regulated in the diaphragm muscle from 4 week old *mdx* mice, and this was associated with increased expression of pSTAT3 [93]. Furthermore, IL6 blockage in *mdx* mice, via neutralizing antibody against the IL6 receptor, improved the muscle phenotype by decreasing inflammatory markers and increasing muscle differentiation and maturation [93]. Other studies have also evaluated STAT3 levels in *mdx* mice. The expression of several transcription factors, including STAT3, was shown to be upregulated in diaphragm muscles from 6 week old *mdx* mice compared to WT mice [57]. Sphingosine-1 phosphate (S1P) is a bioactive lipid that modulates the activity of many different pathways (e.g., AKT signaling and Ras/MAP kinase cascade), and has been implicated in the activation of satellite cells [24,94,95]. In a study evaluating the role of S1P in muscle satellite cell function, results demonstrated that S1P catabolism was associated with lower STAT3 activation and poor satellite cell function [96]. Moreover, TA muscles from *mdx* mice had higher levels of sphingosine phosphate lyase (SPL), the catabolic enzyme responsible for the irreversible catabolism of S1P, and lower levels of serum S1P [96]. Inhibition of SPL increased the levels of S1P in *mdx* mice, thus increasing the levels of pSTAT3 and improving muscle regeneration [96]. Compared to *mdx* mice, dystrophin/utrophin double KO (*mdx*/utr^−/−^) mice have a more severe phenotype characterized by altered neuromuscular and myotendinous junctions, early onset of damage in the diaphragm (as early as 6 days after birth), joint contractures, kyphosis, and premature death [97,98]. Levels of activated STAT3 were also shown to be increased in muscles from *mdx*/utr^−/−^ mice [8]. Moreover, *mdx*/utr^−/−^ mice administered an anti-IL6R antibody (MR16-1) had a decrease in levels of pSTAT3, which was associated with improved phenotype of the limb muscles and decrease in serum CK levels [8]. Overall, findings from these studies demonstrate that IL6 could potentially be used as a pharmacological target in the treatment of DMD. Nevertheless, muscles from *mdx* mice showed different expression patterns of IL6 and pSTAT3, suggesting that while pSTAT3 activation might ameliorate the disease phenotype in some muscles (i.e., TA), it can also be detrimental for other muscles (e.g., diaphragm).

Congenital myotonic dystrophy type 1 (CDM) is a severe type of myotonic dystrophy characterized by hypotonia and muscle weakness caused by the expansion of a CTG repeat in the 3′ UTR of *DMPK* gene. Recent findings demonstrated an upregulation of the IL6 pathway in muscles from CDM patients [99]. Briefly, gene expression profile analysis of affected CDM muscles revealed a significant upregulation of different genes, including serum amyloid A1 gene (SAA1), a gene that is regulated by the IL6/STAT3 signaling pathway [99]. Further analysis demonstrated that members of the IL6 pathway (e.g., STAT3), as well as genes regulated by IL6/STAT3 pathway, were increased in muscles from CDM patients [99]. The increase in the IL6 pathway was not caused by infiltrating inflammatory cells, but rather it was due to increased IL6 production in the muscle itself [99]. IL6 expression correlated with muscle immaturity, which is supported by other studies showing that increased activation of the IL6/STAT3 pathway leads to decreases in muscle mass or increases muscle wasting in different mouse models [42,100].

Muscular dystrophies are genetic diseases characterized by progressive muscle loss and weakness. The underlying mechanisms contributing to MD disease pathology are complex and not completely understood. In here, we discussed the studies demonstrating that pSTAT3 levels are altered and play a role in DMD and CDM. Due to the role of STAT3 in satellite cell function, muscle regeneration, and overall skeletal muscle maintenance, future studies should focus in further evaluating the underlying mechanisms behind STAT3 signaling, including its downstream targets, in different MD.

## 7. STAT3 and TGF-β1 Interplay in Muscle and Other Cell Types

The transforming growth factor-β (TGF-β) superfamily is composed of at least 30 members that control a wide range of cellular functions, including developmental and homeostatic processes [101]. TGF-β signaling has been shown to control the behavior of cells by influencing the rate of cell proliferation and differentiation, as well as tissue homeostasis and responsiveness to damage [101]. Members of the TGF-β superfamily include TGF-β (1, 2, and 3), inhibins, activins, nodal, myostatin, bone morphogenic proteins, and anti-Mullerian hormone [102]. TGF-β activation can be accomplished by several different mechanisms, including increases in levels of reactive oxygen species, changes in pH, activation by integrins, thrombospondin-1, or by proteolytic activation of the latent TGF-β [103]. Once activated, TGF-β binds to its receptor on the cell membrane and activates downstream targets such as SMADs, focal adhesion kinases (FAKs), or mitogen-activated protein kinases (MAPKs), which will enter the nucleus and regulate the transcription of different genes [104]. In this section, studies evaluating the role of TGF-β on STAT3 signaling or vice-versa will be discussed.

TGF-β1, one of the most well-known members of the TGF-β superfamily, was discovered over 30 years ago, and since then has been shown to play a role in a variety of cellular functions [105]. Studies have shown that increased activation of TGF-β1 leads to apoptosis of skeletal muscle precursor cells, endomysial fibrosis, muscle atrophy, and myoblast differentiation into fibrotic cells in vivo [106,107,108]. TGF-β1 is upregulated at the site of muscle injury and is thought to participate in the inflammatory response to muscle damage [109]. Briefly, macrophages are present at the site of muscle injury, and are known to secrete cytokines, including TGF-β1, that will play a role in the process of muscle degeneration/regeneration [109]. However, prolonged release of TGF-β1 at the site of injury is known to contribute to the formation of fibrotic tissue [110]. Nevertheless, in vivo neutralization of TGF-β1 in regenerating muscle reduces the diameter of muscle fibers, which shows that TGF-β1 is required for proper muscle regeneration [111].

There is limited literature on the interaction between STAT3 and TGF-β1. Additionally, while some studies show a direct interaction between STAT3 and TGF-β1, other studies show that STAT3 interacts with the TGF-β1 signaling cascade. Hepatocellular carcinomas (HCC) are a result of liver cirrhosis and advanced fibrosis [112]. STAT3 is a major contributor for the formation of HCCs, since STAT3 has been shown to increase cell proliferation and tumor angiogenesis, while decreasing apoptosis and reducing activation of antitumor immunity [113,114,115,116]. Deletion of the suppressor of cytokine signaling-3 (SOCS3) gene in mice injected with dimethylnitrosamine, a drug known to induce liver fibrosis, resulted in increased activation of STAT3 in liver cells [112]. Moreover, SOCS3 gene deletion also increased the expression of TGF-β1, but this was suppressed by dominant-negative STAT3 overexpression both in vitro and in vivo [112]. Lastly, two potential STAT3 binding sites at the promoter region of TGF-β1 were identified, which shows that STAT3 can induce TGF-β1 gene transcription [112]. Another study has also shown that STAT3 can drive TGF-β1 gene expression [117]. Briefly, hepatitis C virus (HCV) infected hepatoma cells induced the activation of STAT3 and other transcriptional factors involved in TGF-β1 gene expression. Inhibition of STAT3 in HCV-infected cells significantly decreased TGF-β1 gene transcription [117]. More recently, TGF-β activation has been shown to lead to STAT3 phosphorylation in hepatic cells [118]. More specifically, within minutes of TGF-β activation, Janus kinase 1 (JAK1) binds to the TGF-βRI and activates STAT3 in both SMAD-dependent and -independent manners in hepatic cells [118]. The initial TGF-β-induced STAT3 activation primes the hepatic cells for further activation of STAT3, which is potentially driven by cytokines and growth factors [118].

Other studies have also shown different interactions between TGF-β1 and STAT3. Severe acute respiratory syndrome coronavirus papain-like protease was shown to induce a TGF-β1-mediated pulmonary fibrosis via activation of reactive oxygen species (ROS)/p38 MAPK/STAT3/Egr-1 pathway [119]. The activation of the ROS/p38 MAPK/STAT3/Egr-1 pathway led to increases in the expression of TGF-β1, thrombospondin-1, and pro-fibrotic genes [119]. STAT3 can also interfere with the TGF-β1 signaling cascade. STAT3 was shown to affect the TGF-β1 signaling cascade by directly competing with SMAD4 for SMAD3 binding in epithelial cells, thus attenuating the formation of SMAD3-SMAD4 complexes and inhibiting the DNA-binding ability of SMAD3 [115]. Collectively, findings from these studies using non-myogenic cells demonstrate that STAT3 and TGF-β1 have a complex interplay, in which each has the ability to interfere with the other, and the outcome of such interactions depend on the type of cell and environment.

## 8. Evidence of STAT3 Activation by TGF-β1 in Skeletal Muscle

To our knowledge, only one study has demonstrated interplay between TGF-β1 and STAT3 in skeletal muscles. Recent evidence demonstrated that TGF-β1 induced the phosphorylation and activation of STAT3 in muscles from TGF-β1 overexpressing mice [5]. Conditional overexpression of TGF-β1 in skeletal muscles induced muscle atrophy, but not all mice used in the study developed the same disease phenotype [107]. While 50% of the mice developed a milder or no phenotype, the other half showed severe muscle pathology and loss in body weight within 2 weeks of TGF-β1 transgene induction [107]. Findings from the study further demonstrated that levels of pSTAT3 were significantly higher, by both western blot and immunohistochemistry, in muscles from mice with the severe phenotype. C2C12 cells were used to evaluate the effects of TGF-β1 in STAT3 activation in vitro [5]. Results demonstrated that C2C12 cells treated with recombinant TGF-β1 protein had approximately an 8-fold increase in the levels of pSTAT3 (Tyr705) [5]. Overall, this was the first study demonstrating an interaction of TGF-β1 and STAT3 in skeletal muscles from mice [5]. Future studies should evaluate if TGF-β1 and STAT3 might also interact in diseases characterized by alterations in either of their levels, including MD.

## 9. Summary

Recent studies in STAT3 signaling demonstrated its direct role in controlling the myogenic capacity of myoblasts and satellite cells, and the potential benefit of using a STAT3 inhibitor to treat muscle diseases. However, prolonged activation of STAT3 in muscles has been shown to be responsible for muscle wasting by activating protein degradation pathways. It is important to balance the extent of STAT3 activation and the duration and location (cell types) of the STAT3 signaling when developing therapeutic interventions. Figure 1 summarizes the known pathways associated with STAT3 signaling in skeletal muscle as well as non-muscle cells (hepatocytes and keratinocytes). Future studies should evaluate the underlying mechanisms behind STAT3 control of muscle growth and regeneration, including its downstream targets and interactions in different cell types. Multiple therapeutic approaches targeting STAT3 are currently being tested, and this could potentially lead to the development of therapies for MD and other muscle wasting diseases. Nevertheless, due to the role of STAT3 in multiple cell types and intracellular pathways, results from studies should be carefully interpreted.

## Figures and Tables

**Table 1:** List of genes that are directly or indirectly regulated by STAT3. This table was generated by integrating data from seven genome-wide studies of direct target genes of STAT3 by microarray, ChIP or ChIP-Seq [44,50,51,52,53,54,55]. The resulting list was then cross referenced with the literatures to identify the genes that were experimentally proven to be directly or indirectly regulated by STAT3 in the skeletal muscle. ND: not determined.

Gene Symbol	Regulatory Target	Reference
*c-Fos*	Direct	Trenerry et al., 2007 [23]
*Socs3*	Direct	Lieskovska et al., 2003 [56]
*Jun*	Direct	Dogra et al., 2008 [57]
*Myod1*	Direct	Yang et al., 2009 [58]
*Pdia3*	Direct	Burniston et al., 2014 [59]
*c-Myc*	Direct	Srikuea et al., 2011 [60]
*Tlr4*	Indirect	Kim et al., 2013 [61]
*Lif*	ND	Megeney et al., 1996 [62]
